# Dr. Merriman's Synopsis of the Various Kinds of Difficult Parturition, with Practical Remarks on the Management of Labours

**Published:** 1820-12-01

**Authors:** 


***A Synopsis of the various kinds of difficult Parturition, with Practical Remarks on the Management of Labours.*** By Samuel Merriman, M. D. F. L. S. Lecturer on Midwifery; Physician-Accoucheur to the Middlesex Hospital and to the Parochial Infirmary of St. George's, Hanover Square; and Consulting Physician-Accoucheur to the Westminster General Dispensary. Third Edition with considerable Additions and an Appendix of Illustrative Cases and Tables. Octavo, pp. 323. London, 1820.

"Da spatium tenuemque moram, male cuncta ministrat
"Impetus." *Statii Theb. Lib.* 10.

There never was a period, when a work on the obstetric art was read with so much interest, as Dr. Merriman's will be at the present moment. A recent, melancholy event has given rise to an unusual solicitude respecting all difficult and even tedious labours; and the medical republic will rejoice to find that a third edition of the excellent book before us is presented to their attention. The numerous and importunate avocations in an extensive practice of midwifery, together with lectures and consultations, occupy so much time, as to render literary pursuits exceedingly difficult: hence we have fewer volumes written on this, than on the other branches of the profession.

In pursuance of our system of diffusing practical information, we shall endeavour to convey a general outline of the work before us; interspersing such remarks as our own obstetric experience may suggest.

The dedication of a book seldom attracts the notice of a reviewer, who, in general, is anxious to hasten towards the table of contents. In this instance, however, we are induced to do justice to the grateful and affectionate disposition of Dr. Merriman, by informing our readers that his book is not ushered into the world under the splendid auspices of a noble lord, but is simply dedicated to the memory of his late uncle, Dr. Sam. Merriman, whose excellent qualities are justly and piously recorded. In a neighbouring nation we believe this custom is common: the celebrated Xavier Bichat addresses his Treatise on the Membranes to his father, whom he called his best friend.

The various kinds of difficult parturition are arranged by Dr. Merriman in a nosological form. This attempt is not new: Sauvages, Sagar, Linnædus, Macbride, Young, and others, include *Dystocia* in their respective systems; and under the genus parodynia Mr. Good has compressed different species and varieties. It has been the object of the author to conform, as much as possible, to the models of Sauvages and Young; and to exhibit a more correct and extensive view of difficult labours, than is to be met with in their works. The arrangement adopted in the book before us, consists of two classes, which are divided into orders, as follow:—

Class I. Eutocia.

| | | | |
|---|---|---|---|
| *Order* | 1. | Eutocia simplex. | *Natural Labour.* |

Class II. Dystocia.

| | | | |
|---|---|---|---|
| *Order* | 1. | D. diutina. | *Lingering Labour.* |
| —— | 2. | D. anenergica. | *Powerless Labour.* |
| —— | 3. | D. perversa. | *Malposition of the Head.* |
| —— | 4. | D. amorphica. | *Deformity of Pelvis.* |
| —— | 5. | D. obturatoria. | *Obstructed Labour.* |
| —— | 6. | D. ectopica. | *Displacement of the Uterus.* |
| —— | 7. | D. transversa. | *Preternatural Presentations.* |
| —— | 8. | D. gemina. | *Twin Births.* |
| —— | 9. | D. laceratoria. | *Ruptures and Lacerations.* |
| —— | 10. | D. hæmorrhagica. | *Flooding Cases.* |
| —— | 11. | D. syncopalis. | *Faintings and Palpitations.* |
| —— | 12. | D. epileptica. | *Puerperal Convulsions.* |
| —— | 13. | D. inflammatoria. | *Fevers and Inflammations.* |
| —— | 14. | D. retentiore. | *Retention of the Placenta.* |
| —— | 15. | D. inversoria. | *Inversion of the Uterus.* |

This division appears sufficiently minute; but we think it would have been more consistent with the rules of classification to have thrown the orders into species, and to have adopted Mr. Good's class *genetica*, and order *carpotica:* thus making eutocia and dystocia two genera, instead of two classes, and the subdivisions in the sections, varieties instead of genera.

On the management of *lingering labours* arising from original or accidental weakness of habit in the mother, the author makes the following remark.

"If there be want of rest, from ♏x to ♏xx of tinct. opii, may be given with great advantage. Much larger doses of opium, namely, to the extent of six, eight, or ten grains of *extr. opii*, have been recommended in this kind of slow labour, with a view to relax spasm, and render the uterine action more perfect; but such herculean doses can very rarely be necessary, and would not always be safe." P. 25.

In a note he observes that three cases have occurred within his own knowledge, in the practice of a midwife, where an entire suspension of uterine action was produced, requiring the aid of instruments to effect delivery, by, as he conceived, the injudicious exhibition of this medicine.

An *irregular*, *partial* action of the uterus, excessively *painful*, and often *premature*, has frequently come under our notice, and has been speedily removed by two or three grains of opium. We have known instances of its continuance for the space of several days, during which time, no impression has been made on the os uteri. On one occasion we were called in by a surgeon, who had been in attendance three days, until his patience was exhausted, in expectation, hour after hour, of some progress being made in, what he had supposed to be, labour. We directed three grains of opium, and the patient was speedily relieved. Regular labour did not follow in less than a month. Cold, fatigue, and sometimes intestinal disorders, have appeared to us to have been the remote causes.

In *tedious labours*, it was the practice of Mauriceau, to administer an acidulated infusion of senna, and to inject a stimulating clyster. We are ourselves partial to the latter remedy, and remember once to have observed the most rapid and vigorous uterine action produced, and the largest fœtus we ever saw expelled, by its use in a case of very tiresome labour.

When *fever* of an *inflammatory* type is present, bleeding is exceedingly beneficial; but it is a remedy requiring caution in its employment.

The membranes, Dr. Merriman remarks, should not be artificially ruptured:—

"1. While the head of the *fœtus*, or a large portion of it, is above the brim of the pelvis.

"2. While the *os uteri* is undilated, or in a state of rigidity.

"3. While the perinæum is thick and firm, or rigid." P. 33.

When the fœtus has been long dead, the abdomen, being greatly distended, with putrid air, should be punctured. The author has known two fatal instances of rupture of the vagina, which arose from a neglect of this precaution, and from the rashness of the midwives.

It is necessary to pay particular attention to prevent a laceration of the perinæum, when the *forehead* is inclined towards the *pubes*; and when the *face* is the presenting part, although the expulsion may be generally left to nature, yet under certain, peculiarly favourable circumstances, turning of the child might probably be adviseable. In face-presentations there is perhaps a peculiar tendency to retention of urine, which must be carefully obviated.

The following passage on *dystocia amorphica* cannot be too strongly impressed on the minds of all accoucheurs:

"It becomes us to be exceedingly cautious, not to suppose, upon light and insufficient grounds, that the distortion is too great to allow the child to pass without the intervention of instruments; and particularly when there is a question about employing the *perforator*, an instrument always incompatible with the life of the child, we ought to weigh every circumstance very carefully in our minds, and, *if possible procure the opinion of some other experienced practitioner*, before we determine upon having recourse to it.

"The maxim of the stern satirist,

*Nulla unquam de morte hominis cunctatio longa est,*

applies not only to the adult, but to the infant in the womb." P. 49.

In proportion as surgeons are better educated, they become more conscientious and humane; and we trust, ere long, that the barbarous operation of embryulcio will be found to be very rarely *necessary*. Placed in situations affording most extensive practice in midwifery, and having been often called upon to assist others, we have never in the course of the last 20 years, found it requisite to open the head, or to employ any other instruments in difficult labours than the forceps or lever. We have repeatedly been requested to perform the operation by those, who have believed the child to be dead and the mother incapable of being relieved by any other expedient. Even in these instances, by the judicious use of the lever, we have delivered the patient in an hour or two of a *living* child. Moreover, we have attended one woman, who, at an advanced age, brought a fine, living child into the world without any artificial aid, after she had formerly several times submitted to the unnecessary destruction of her infant, and subsequently to the operation for inducing premature labour. By want of sleep, and by anxiety and uninterrupted attention to one subject, the mind becomes fatigued and loses its healthy tone; like a spring which has been over-bent. Hence it is not uncommon for the medical attendant to experience a temporary loss of, what Cicero calls, the "robur animi"; and his judgment being thus impaired, he is not, in our opinion, so fit to decide on the propriety of the important operation, we are speaking of, as when he first entered the apartment of his patient. To a person so circumstanced, the comfort and advantage resulting from the friendly advice and co-operation of an experienced, professional friend, are abundantly apparent.

The symptoms denoting the *death* of the child, both before and after labour has commenced, are enumerated by Dr. Merriman with sufficient minuteness; and he concludes his remarks by observing that

"We ought never to be satisfied with a single token of death; but should examine each symptom separately, and afterwards several collectively, before we allow ourselves to come to the ultimate conclusion." P. 55.

*Dystocia obturatoria* may be occasioned by the presence of the hymen, or by a cohesion of the labia, or of the vagina; but the action of the uterus will alone be sufficient in most cases to overcome the difficulty.

*Tumours* growing from the organs of generation may require the aid of the forceps or vectis; and, should these be inadmissible, it would be right to pause, and to consider whether to remove the tumour, or to diminish the size of the child, would be most likely to be attended with ultimate advantage. In three out of five cases, in which the perforation was employed, the mother did not recover; and when turning was undertaken, it does not appear to have been a successful mode of practice either to the mothers or the infants. When the tumours have been opened, it has in several instances been afterwards necessary to perforate the fœtal head. The evidence we at present possess is more in favour of opening the tumours, when they contain a fluid, than of any other mode of procedure. The Cæsarian operation has never been attempted, we believe, in labours of this kind; and its general fatality in this country might be a very great objection to it. A plate, representing an ovarian tumour, which prevented the descent of the child's head, is annexed.

Protrusions of the urinary bladder into the vagina may be detected and cured by the catheter.

In cases of *dystocia ectopica*, occasioned by a projection of the os and cervix uteri, without the os externum during labour, the os uteri should, if possible, be returned within the vagina, and the edge of it ought to be supported by the expanded hand during the passage of the head.

That the os uteri is sometimes found projecting above the symphysis pubis was explained in a dissertation by Dr. Merriman in 1810; and his uncle and Dr. Denman were both convinced of the fact in a patient whom they attended. This opinion has also been subscribed to by several other practitioners. But the author says he is

"Informed that a professor of midwifery, whose practice and lectures at a celebrated university have ensured him a high reputation, asserts the utter impossibility of such an occurrence, which, according to him never did, and never will, happen." P. 64.

*Breech-presentations* were formerly supposed to require the operation of turning the child; and even Dr. Hunter, in the beginning of his practice, adopted the old doctrine of pushing up the presenting part and turning. At that time he lost almost every child, but after he left the process to nature, he always met with success. A fillet may be useful, passed between the belly and thighs; but the blunt-hook should never be applied, as Dr. Young says, it is difficult to avoid breaking the child's thigh. Professors Plenk and Wrisberg, and Dr. Hamilton recommend the forceps to be employed; but our author has had no experience with them in nates-cases, and in the *London Practice of Midwifery*, which is supposed to exhibit the doctrines of a celebrated teacher now deceased, they are represented from frequent trials to be of no use.

*Presentations of the inferior extremities*, by occasioning a compression of the funis, expose the child to considerable danger. The delivery of the body should be effected slowly, and care should be taken to conduct the forehead into the hollow of the sacrum. The same rules apply where the nates present. In these cases, an ample dilatation having been effected by the expulsion of the nates, the arms should be brought down; but in feet-presentations, it will be better to let them remain, as there is danger of fracturing or dislocating them, on account of the contraction of the os uteri round the upper part of the head, especially where the birth of the child has been hurried by turning. The head should be extracted with all the speed which circumstances will admit. For this purpose a finger of the left hand should be introduced into the child's mouth, and the fore and middle fingers of the right hand should be passed over the nape of the neck, one finger resting on each shoulder. A moderate, extracting force may now be carefully employed to bring forth the head. The force should be made upwards and downwards, or from side to side. We remember an instance in which, from ignorance or carelessness, a *rotatory* motion was exerted to such a degree, as to separate the body from the head; and the means attempted to remove the latter were so awkward, as to occasion the mother's death.

There are no presentations more difficult to manage, or more dangerous, than those of the *upper extremities*. From the time of Ambrose Paré to the present, it has been generally agreed that the best practice is to turn and deliver footling. This is most easily done, when the os uteri is sufficiently dilated, before the membranes are ruptured; and care should be taken to bring down the feet along the belly, not over the back of the child. When the arm has descended into the vagina, it is seldom that we are able to take hold of both feet. If the liquor amnii has been evacuated, and the os uteri but little dilated and rigid, the safer plan will be to wait until the parts become more relaxed, for fear of exciting inordinate or spasmodic action, or a laceration of the uterus. Indeed, in all cases, when the os uteri is firm and rigid, it will be found best to wait with patience; as we have known dangerous and even fatal consequences arise from too hasty a dilatation under these circumstances. We have seen cases of this kind attended with a quick, hard pulse, furred tongue, and other inflammatory symptoms, in which we have thought bleeding at the arm might have been advantageous. This practice has been adopted with the best effects, when the labour-pains recur often and inordinately strong and forcing. Dr. Hamilton recommends the exhibition of eighty drops of laudanum. The os uteri having become more yielding and the excessive, uterine action having subsided, we must proceed to deliver without delay. By this cautious mode of proceeding it has often happened that the lower extremities have been brought down by a spontaneous evolution, a phenomenon first accurately noticed by Dr. Denman. Dr. Douglass of Dublin believes, that this natural process would more frequently take place, if it were more frequently trusted to by the obstetrick attendant. There does not appear to be any advantage in one position of the patient more than another, but Dr. Merriman has sometimes thought that he has facilitated the passage of the head through the pelvis by placing her on her back.

In the practice of Dr. Merriman and his uncle, amounting together to nearly 20,000 labours, no instance has occurred of presentations of the back, belly, or side.

It was formerly supposed, when the *funis* descended into the vagina, that the child lay across the pelvis, the abdomen being over the os uteri. The head, the *nates*, or one of the extremities will be found presenting: the belly being seldom or never in that situation. While the funis continues to pulsate, there is hope that the child's life may be saved; and various contrivances have been suggested and tried for the purpose of returning the funis and retaining it above the head, and out of the sphere of compression. Any of these means may be attempted, but as they frequently fail, the best practice is to turn and deliver by the feet, if the following favourable circumstances, enumerated by Dr. Haighton in his syllabus of lectures, are present.*

" 1st. A pulsation of the cord, proving the life of the child;

" 2d. The head not having yet entered the pelvis;

" 3d. Pains not strong;

" 4th. A relaxed state of the external parts, to admit of a ready extrication of the head."

Should the arm and funis present together, turning will be indispensable.

In *twin*-cases, is the birth of the second child to be left to nature, or terminated by art? The following is an outline of Dr. Merriman's practice.

" 1. When both the children present naturally, and the labour of the first terminates without artificial assistance, and without much fatigue to the patient, I wait for the spontaneous occurrence of the secondary pains; but should these not come on soon, or in a *reasonable* time, I rupture the membranes; and then commonly find, that the second child passes with comparative ease through the pelvis, the parts having already undergone sufficient dilatation.

---

* Dr. Ducamp, of Paris, has invented a very ingenious contrivance for returning the funis umbilicalis, a drawing of which we hope soon to present in this Journal. It is now in the hands of Mr. Alcock for that purpose. *Ed.*

"2. If the first labour has been natural, and the second child presents in a wrong direction, I have deemed it generally expedient, with very little delay, to extract it by the feet.

"3. If the first labour has been preternatural, or very difficult, or dangerous, this has always seemed to me an additional reason for terminating the second, as expeditiously as circumstances will admit. Whether, in this case, it will be sufficient merely to rupture the membranes, or whether it will be preferable to bring down the feet, or to assist in any other manner, the accoucheur in attendance must determine." P. 120.

*Lacerations* are divided by our author into five species: laceration of the perinæum, of the labia pudendi, of the vagina or uterus, of any other internal organs, and of the ligaments of the pelvis. Laceration of the perinæum is a most distressing accident, especially when it extends into the rectum; and when it has once occurred, we have found it liable to happen during subsequent labours, unless prevented by great care. Besides a due support of the perinæum during the expulsion of the head, several preliminary measures are recommended with much judgment. The cure of this misfortune has been attempted by sutures, but, as might have been expected, without success. In parts, so loose and flabby, the smallest wound is apt to produce the suppurative inflammation; and the kind of wound produced by parturition is a torn, not an incised one. Great pains have also been taken with adhesive plaster, but we need not say that they were ineffectual. The plan tried by Smellie was rational, and we think might succeed in some favourable cases, if carefully conducted. He waited till the parts were cicatrized, and the patient had recovered from her confinement, when he performed an operation somewhat similar to that for the hare-lip. The treatment we have adopted has always been to poultice in the first instance, and, after suppuration has been established, and the tumefaction has subsided, to insinuate between the lacerated parts a little lint immersed in a lotion composed of tinctura myrrhæ and liquor calcis, in the proportion of one part of the former to five of the latter. Our patients have all done well; and lacerations of the labia pudendi have been always cured in the same manner.

Lacerations of the vagina and uterus have happened from a morbid state of the parts, but the most common cause is the violence of the labour-pains acting irregularly, or impetuously against some projecting part of the child, upon which the uterus splits. This is most likely to take place, when the pelvis is distorted, or the presentation is preternatural. It may be occasioned by the operation in turning the child, or in applying instruments; and sometimes the bulk of an emphysematous child, in passing through the os uteri and vagina, has torn these parts.

*Rupture of the uterus* may take place before the full term of utero-gestation, and while the os uteri is undilated. Here it is impossible to afford any manual assistance, excepting by the operation of *gastrotomy*, which we think would be an uncertain expedient. In a few instances, at the expiration of months or years, the child has been removed in a dissolved state by a process of ulceration established by Nature.

When this accident happens during the pains of labour, these symptoms speedily present themselves; a sense of something giving way internally, preceded by a severe pain, described as cramp; great languor and debility; a sudden vomiting of the contents of the stomach, also a vomiting of a brownish or coffee-coloured fluid; a quick, weak, fluttering pulse; cold perspiration; dyspnœa; an instant cessation of the labour-pains. The presenting part, being retracted, is no longer within reach of the finger; the child is generally discovered to have escaped into the abdomen, but in a few instances has remained in the uterus after the laceration. It has been recommended to pass the hand through the rent and to deliver by the feet; and it has succeeded in saving the patient's life; but in general all that can be done proves unavailing. This practice was countenanced by Dr. Denman; but in his *Observations on the Rupture of the Uterus*, published in 1810, he seems to be convinced that the patient would often have a better chance of recovery, were the case left to Nature, than by any interposition of art. When the child has only in part left the uterus, Dr. Merriman considers it the best practice to deliver footling or with the forceps, according to the presentation; and if the lacerated part be of sufficient extent, and the accident be very recent, he believes it would be expedient to deliver by the feet, even should the child have entirely escaped from the uterine cavity.

*Rupture of the urinary bladder* can only happen from neglect of the practitioner, who should be careful to examine the abdomen, and introduce the catheter from time to time, if needful. We shall extract the following paragraph from our author's book, for the purpose of making a remark upon it.

"Should the laceration allow the urine to escape into the cavity of the abdomen, there can of course be scarcely any expectation of a recovery, but sometimes the laceration has been at the *cervix vesicæ*, opening into the *vagina*; this accident is not necessarily fatal; but the patient will ever afterwards remain in a most uncomfortable state, from a constant involuntary discharge of urine." P. 111.

The eneuresis consequent on difficult parturition, to which we suppose Dr. Merriman alludes, we have always found to proceed from pressure too severe and long-continued for the parts to bear, and not from laceration or rupture. There will, in fact, be found a loss of substance occasioned by the *sloughing process*, following high inflammation. In whatever way a communication is formed between this viscus and the vagina, the surgeon ought not, in our opinion, to consider the case incurable: for when he is called in a few days after the sloughs have been separated, or before cicatrization has been completed, we should have very sanguine expectations of a perfect cure being accomplished by the adoption of the ingenious plan of treatment pursued with success by Mr. Barnes,* which reflects much

---

* See Medico-Chirurg. Transactions. Vol. vi, page 583.

credit on his judgment and humanity. We should not entirely despair, were we consulted after the fistula had been formed a considerable time; since we find that, under these circumstances, Desault effected cures by means of pressure continued with great patience and perseverance.

The aorta, internal iliac vein, the liver and other viscera, have been ruptured during labour.

"When great numbness in the lower extremities continues for a considerable time after delivery, with inconvenience and difficulty in moving the thighs, and pain and tenderness about the groins or hips, it may be supposed, that a laceration of the ligaments of the pelvis has happened in a slight degree. More rarely a greater degree of laceration befals these parts, for sometimes the bones of the pelvis are forcibly separated, producing a state of lameness and weakness, which months and years very imperfectly overcome." P. 112.

Labour attended with *hæmorrhage* requires nice management. The patient should be kept quiet, cool, and recumbent. Cold clysters should be administered. The diet to be simple and limited. Stimulants to be avoided. Refrigerants internally and cold liquids or ice externally. Venesection to be used cautiously, and in placental presentations and hæmorrhage after delivery inadmissible.

"And the *plumbi acetas*" (superacetas), "and many other internal remedies, which are efficacious in restraining chronic hæmorrhages, cannot be at all relied upon in the floodings of parturition." P. 113.

Dr. Merriman divides these hæmorrhages into three species: accidental, unavoidable, and atonic. The first occurs when the placenta is naturally situated within the uterus, and is separated by accident. A considerable discharge of blood may take place within the uterus, occasioning syncope or death, with very little appearance of discharge from the vagina. The alarming symptoms we apprehend arise from the rapidity with which the hæmorrhage occurs, and not from the quantity of blood poured forth: for in a lady, attended by Mr. Saumarez, who died from this accident, only a pint and half of blood were found extravasated on dissection. In addition to the other remedies, diluted sulphuric acid may be given; but bleeding should not be employed, unless the pulse be very hard, strong, and active. If the action of the uterus should commence, and the flooding in consequence diminish, farther means may be unnecessary; but if these events should not happen, it will be proper to introduce the finger, and, having dilated the os uteri, to rupture the membranes during pain.

The hæmorrhage, proceeding from an *attachment of the placenta over the cervix uteri*, was not generally understood till of late years. Instead of being originally deposited in this situation, it was formerly supposed to have dropped down by its own weight; and the opinions of Portal on this subject, published in 1685, were strangely overlooked by his contemporaries. This species of flooding is increased by every pain, which, by promoting the dilatation of the os uteri, lacerates the placenta from it. In all cases of this nature, to turn and deliver by the feet will be indispensable. Nature may now and then be sufficient without assistance, but such a rare occurrence must not induce us to interfere with an established rule of practice, particularly one of such vital importance as this. When the hæmorrhage is profuse, no time is to be lost; but when it is moderate and no particular expedition is called for, we perfectly agree with Dr. Merriman that it is desirable to obtain, if possible, a certain degree of softness and dilatability of the uterus. The os uteri is sometimes found thick, unyielding, and exceedingly tender. In this state, a rude dilatation would be hazardous to the mother, and difficult to the operator. A gentle hæmorrhage here does good, by unloading the turgid vessels, and, if we may be allowed the expression, by removing the inflammatory state of the parts. The operation is performed either by breaking down the adhesions between the placenta and cervix uteri, rupturing the membranes and taking hold of the feet; or by perforating the placenta with the fingers, and thus getting in contact with the body of the child. The former, if practicable, appears to be the most advisable process. Every step must be taken with firmness and deliberation, and the patient's strength supported by proper nourishment. The child and placenta being removed, the practitioner should be satisfied that the uterus has duly contracted itself. Dr. Merriman has often observed the *phlegmatia dolens* to follow.

All the usual means of suppressing hæmorrhage are to be employed when it takes place *after delivery*. If the placenta is still retained, the hand must be introduced to separate it; but if expelled, a broad bandage should be put round the body, and a sponge immersed in port-wine, cold vinegar and water, or a lump of ice may be introduced into the vagina. Large coagula, preventing the contraction of the uterus, should be withdrawn by the introduction of the hand.

On the practice of applying plugs in uterine hæmorrhage some sensible remarks are made by the author, who says that they are inapplicable when the bulk of the uterus exceeds that of a pregnancy of three or four months, and when the parietes are easily distended. The indiscriminate use of opium is also condemned. A case is related by Dr. Atkinson, in which 80 drops of laudanum so far paralyzed the uterus, as to render it incapable of farther contraction, and the patient died. Dr. Merriman remarks,

"The cases in which it" (opium) "seems most beneficial, are states of irregular, spasmodic action of the *uterus;* cases requiring the child to be turned, but where the rigidity of the *os uteri* prevents the ready introduction of the hand; and cases, where, after delivery, great irritability prevails." P. 128.

The *ergot* of rye has been recommended in hæmorrhages, and in sudden deaths the transfusion of blood; but Dr. Merriman has had no experience of their efficacy.

In women of a delicate frame, of a nervous, irritable, and hysterical habit, *fainting, a sense of distress and oppression about the præcordia, and palpitations* are apt to take place. These complaints also happen to women exhausted by fatigue, by want of food and of sleep, and by apprehension or any other debilitating causes. They are mostly of an hysterical nature, but when preceded by organic diseases, they are of a more serious and alarming nature. The remedies recommended are, camphor-julep, sp. am. arom., sp. æth. sulph. &c. and opium, if great fatigue or want of sleep should be the cause. When the fainting is of long continuance or frequently repeated, it would probably be needful to expedite the delivery by any *safe* method, and the same may be said of fainting when it proceeds from exhaustion.

Labour accompanied with *epileptic fits* has always been considered very dangerous; but the proportion of deaths in modern practice is not so great as it was formerly. Dr. Merriman very properly confines the term convulsions to those fits, which bear an exact resemblance to epilepsy. The etiology of the disease has been variously stated. Those who have believed it to be produced by *general* irritability, have advised opium; by irritability of the *uterus*, immediate delivery; by an overloaded state of the system, large bleedings and other evacuants. Dr. Merriman never saw a case, in which at the commencement, he could have dared to give opium; and Dr. Hamilton always found it prove fatal at this period.* With respect to turning, the author does not have recourse to it on all occasions; but he strenuously recommends the adoption of the depletory plan of treatment.

"In most of the cases that I have seen, the evacuations from the bowels produced by cathartics, have been dark coloured, heavy, copious and very fetid: I hardly recollect an instance in which the blood has not shewn an inflammatory crust; and it has often been very much cupped.

"These facts will, I conceive, authorize me to recommend, in the first instance, having recourse to the depleting plan." P. 136.

On the first occurrence of the fits, whether before, during, or after the labour, from 10 to 20 ounces of blood should be drawn from the arm, jugular vein, or temporal artery; from 5 to 10 grains of hydr. submur. either in a pill or mixed with moist sugar, should be exhibited; and a solution of salts once in three or four hours, until stools are procured. If the stomach should be overloaded, vomiting may be excited. The head to be shaved and constantly covered with the following lotion:

℞. Liq. am. acet. f. ℥vj.
Sp. rorism. f. ℥ij.
Aquæ puræ, Oj. M.

---

* *Annals of Med.* vol. v. p. 340.

If the patient cannot swallow, a cathartic clyster to be injected; and should the convulsions continue unabated, it may be necessary to repeat the bleeding generally or locally, according to the state of the pulse.

The labour proceeding quickly, it may not be necessary to do any thing more; but should it be slow, it will be right to apply the forceps and finish the delivery. The perforator is to be avoided, if possible.

"I have so often had the pleasure, by delaying this *dreadful* operation, of seeing my patient delivered of a living child, that I cannot too much insist upon caution, and due deliberation upon this subject." P. 139.

When the convulsions continue after the delivery, the lotion must be persisted in; a blister applied to the back or insides of the thighs; sinapisms to the feet; and, if the patient can swallow, diaphoretics, aperients, and light cordials should be given. The catheter to be used, if needful, twice daily. Dr. Merriman has known two or three cases, which have terminated in temporary mania; and one in true chronic epilepsy, which continued some years, till the patient died of a pulmonic complaint.

The post mortem appearances, noticed by Dr. Denman, were an unusual flaccidity of the heart and an emptiness of the auricles and ventricles, which have also been seen by others. In one instance, the author found an effusion of blood in the posterior part of the cranium.

A table, exhibiting the practice pursued in 36 cases, is subjoined.

The treatment of *fever* or *inflammation*, accompanying pregnancy or labour, must be conducted as circumstances require.

Different opinions have existed respecting the management of the *placenta, when it has been retained an unusual time after the birth of the child.* Dr. Wm. Hunter, for a long time, strenuously recommended its expulsion to be left entirely to nature, but several fatal accidents resulting from this practice, he was induced towards the latter part of his life to alter the opinion he had formed. Experience has now taught us, that the best plan, if the placenta is not expelled within half an hour, is to lay the hand on the abdomen, and gently to rub or press the parts over the uterus. These gentle means not succeeding within an hour or two, we must proceed to extract the placenta; and in cases of hæmorrhage, we must do so immediately.

The introduction of the hand will sometimes produce the effect we wish, if not, the fingers should be moved about gently near the os uteri. When it is necessary to separate the placenta, we must take care to do it completely; and then wait to give the uterus an opportunity of contracting. The whole process must be conducted slowly.

As retention of the placenta has occurred nearly five times as often in Dr. Merriman's public, as in his private, practice, it is to be inferred that it might often be prevented by observing the judicious directions of Mr. White, respecting the expulsion of the body of the child.

*Inversion of the uterus* may happen spontaneously, or from rude attempts to hasten the separation of the placenta. The uterus should be immediately replaced by the introduction of the hand and arm, well defended with lard. When the placenta is attached to the inverted uterus, the latter should be replaced, before the separation is effected. It must be observed that if the re-inversion does not take place before the uterus has contracted, it will be almost impossible to effect it, and the disease will remain incurable, excepting by the operation of excision.

Respecting the use of *instruments in widwifery*, "the old adage," says Dr. Merriman, "*neque temere neque timide*," though trite, is still the best motto, by which the accoucheur can be guided." P. 153.

After carefully examining the respective merits of the forceps and lever, the author seems to prefer the former.

"The judicious practitioner will use sometimes one and sometimes the other, as circumstances may require; but were we compelled to select one only of these instruments for constant use, the *forceps* is that, to which the most decided preference would be given." P. 155.

"So careful have the best professors of midwifery been to guard against an improper use of their instruments that it has been laid down as a rule of practice, that the forceps shall never be applied till the ear of the child has been within reach of the operator's finger at least six hours." P. 156.

To this rule hæmorrhage and convulsions are exceptions.

Dr. Merriman's directions for the application of the forceps and lever do not differ from those laid down by Dr. Denman.

We perfectly agree with the author that the *legitimate* cause for using the *perforator* is *distortion* of the pelvis. This should be constantly borne in mind.

Before we proceed to the operation of cephalotomy, a considerable time should be allowed to elapse; because the operation will be more easily and safely performed; we shall know or believe that it was not done prematurely; and we shall be able to convince the patient and her friends of the necessity of it. *We* form our judgment from the dimensions of the pelvis; *they* from the length and severity of the labour.

To describe the operation here will be superfluous. Indeed we cannot too often repeat that more skill is required in deciding upon the *propriety* of operating, than in performing an operation.

The *Cæsarean operation* in this country has been exceedingly unfortunate; twenty-one out of twenty-two patients having perished. The fate of the children has been more favourable. In Ireland a singular case occurred. The operation was performed with a razor, by Mary Dunally, an ignorant midwife, and the poor woman recovered in twenty-seven days. On the Continent it has been more frequently practised, and been more successful. Our comparative ill success is attributed by Dr. Merriman to previous disease, occasioned by *mollities ossium*, and to the fatigue, languor, and fever resulting from improper delay. The interference of the ecclesiastical authorities in France and their decisions have promoted and familiarized the operation through the French nation; and in Sicily an edict is now in force, declaring that no person shall be admitted to practise surgery, till he has been minutely examined touching the mode of performing the Cæsarian section. The encouragement thus given has led the Continental surgeons to act rashly on many occasions, as Baudelocque himself admits.

In the event of a woman dying undelivered near the expiration of her pregnancy, this operation ought always to be had recourse to without loss of time.

Of the various means suggested to obviate the necessity of piercing the mother's womb at the hazard of her life, or of utterly destroying the child, the most rational and effectual has been found the process of inducing *premature labour.* The section of the *symphysis pubis*, the discovery of which was so pompously proclaimed, is now universally laid aside; and the uncertain and inadequate effect of *abstinence* in retarding the growth of the *fœtus in utero*, recommended by Mr. Lucas, must render that measure inapplicable in cases of distorted pelvis.

Thirty-three cases of labour, brought on artificially in the eighth month, have occurred in Dr. Merriman's practice, or come within his knowledge. Nearly one-third of the children have been saved, and all the women recovered. Certain limitations and cautions are transcribed from a sensible paper published by the author on the subject of inducing labour prematurely, and inserted in the Medico-Chirurgical Transactions, Vol. iii. p. 144.

In an *appendix*, which occupies more than one-third of the volume, Dr. Merriman has brought together a mass of practical information, interspersed with cases, illustrative of his synopsis, which will be found highly useful both to the student and the experienced practitioner. The limits we had allowed ourselves will not permit a minute analysis of this portion of the work; we can therefore only notice a few of the most important articles.

The *clavus*, or *ergot of rye*, *secale cornutum*, or *spurred rye*, has been strongly recommended by some eminent American accoucheurs for the purpose of exciting and invigorating the action of the uterus during labour. A drachm, infused in three or four ounces of boiling water during ten or fifteen minutes, is the usual dose. Should not sufficient pain come on in half an hour, it may be repeated. Dr. Merriman had great difficulty to procure the ergot of rye; but was at length supplied with some by Mr. Henry Davies of Conduit Street, who had received it from Dr. Bibby of New York. The period for its use is when the head has passed the brim of the pelvis. If given before this time, by increasing uterine action, the life of the child, Dr. Bibby says, will be almost certainly sacrificed. It has no effect, when the fœtus has been some time dead. Six cases are related, in which it was administered, but we are sorry to remark, that the results were by no means favourable: three of the children being still-born, three dead, and only one, a twin, born alive.

Some judicious remarks are made by Dr. Merriman on the treatment of those labours, in which the head of the fœtus is distended by *hydrocephalus.* A melancholy mistake was made by a surgeon in one instance, which rendered the woman miserable all the rest of her life. The supposed hydrocephalic tumour was the distended bladder, which, being punctured, became so inflamed that part of it sloughed away, leaving an incurable aperture.

A most extraordinary case of sudden death is related, which was occasioned by violent mental emotion, during labour. This shews the importance of preserving the mind in a state of perfect quiescence.

Baudelocque and another eminent lecturer on midwifery, have stated their belief, that no *pulsation* is to be felt in the *fontanelle* before birth. Our author having, however, carefully examined this part in a fœtus, whose forehead presented favourably during its descent through the pelvis, was fully satisfied six several times that a distinct pulsation was perceptible. As the head descended lower, the pulsation became interrupted, and just as it was emerging, the phenomenon was no longer observable. The child was born alive.

In the next section of the Appendix two cases are recorded, in which the *hymen* was found entire during labour. In the one, the membrane was ruptured naturally by the head of the child: in the other, it was artificially divided: both did well.

Next we are presented with a most excellent account of *polypous tumours*, discovered during labour. In one instance, where the tumour was not removed, the patient, having been delivered, died from excessive pain, occasioned by the constant efforts of the uterus to expel the disease. To these cases we shall add one, communicated to us by Mr. J. M. Coley, of Bridgnorth.

" 1813, *June 22d.* Mary Head, æt. 38, mother of sixteen children, all of whom had been expelled about the 6th, 7th, or 8th month of pregnancy, either dead or nearly so, had complained, during three years, of pain in the right hip and ovary, and in the back. She had frequent rigors, syncope and a copious discharge of blood, alternated with leucorrhœa to an excessive degree. On examination he discovered a polypous tumour two inches in length, and, near its base, one inch in diameter, growing from the inner surface of the cervix uteri. The os uteri was dilated by the tumour to the extent of an inch. An operation being determined, Mr. Coley obtained the able assistance of his father, whose judgment has been matured by a laborious and successful practice of three and forty years. The patient being placed on her left side, Mr. Coley took hold of the tumour with two fingers, and secured it in a noose made with a double ligature, passed through the upper loop of an iron wire introduced as a conductor. The wire, acting as a third finger opposed to the two that had been before passed, was found useful in adjusting the noose round the base of the polypus. The ligature was now drawn quite tight, and secured to the lower loop of the wire; and they were both left in the vagina.

" *24th.* The ligature was tightened.

" *26th.* The tumour came away in a shrivelled state. The os uteri had contracted and a prominence was perceptible on the inner margin of the posterior labium, the whole of which was thicker than the other.

"1814, *July 3d.* State of the os uteri about the same as it was just after the operation. The woman had another child afterwards."

Cases similar to this will be found related by M. Gasc, in Desault's Parisian Chirurg. Journal, Vol. i. p. 278, and by Dr. Denman, in Med. and Phys. Journal, Vol. iii. p. 1. In Med. Chirurg. Transactions, Vol. iii. Drs. Denman and Clarke have published cases and observations with the view of improving and promoting the means of distinguishing the various uterine tumours.

Dr. Merriman gives an account of an *ovarian tumour*, which, obstructing labour, was punctured, and the delivery being afterwards finished with the perforator, the woman died on the fifth day, apparently from exhaustion; no organic disease, excepting that in the ovarium, being discovered on dissection.

In the section on projection of the *os uteri above the symphysis pubis*, the following passage occurs:

"These cases I conceive establish the fact that a retroversion of the *uterus* may exist at the full term of utero-gestation, and, if I mistake not, go far to prove, that many cases on record, of supposed extra-uterine gestation of the *ventral kind*, were in reality cases of retroverted *uterus*. There is something so repugnant to all that we know of the processes of conception and generation, in supposing that an *ovum*, accidentally conveyed into the cavity of the *abdomen*, can there be lodged and nourished, and brought to perfection, altogether unconnected with the uterine system; that unless the fact be clearly and unequivocally proved, it will hardly be believed." P. 235.

We think every one, who will reason on this subject, will be of Dr. Merriman's opinion with respect to those fœtus capable of supporting life; but we believe that many cases have really occurred of imperfect fœtus, or such as were not adapted to support life, which had never entered the uterus, but remained encysted during life, or had been expelled through different parts by the process of ulceration. A remarkable instance of imperfect fœtation is related by our friend Mr. J. M. Coley in the Edinburgh Medical and Surgical Journal, Vol. vi. p. 50. This patient, having died from mortification in the omentum in the fifth month of pregnancy, afforded him an opportunity of ascertaining that the jaw-bone, teeth, and hairs, found partly in the ovary and partly in the intestine, had never entered the uterus; but that the fœtus, with which she was pregnant at the time of her death, was in its natural situation.

When the uterus is strongly contracted in *funis-presentations*, it is exceedingly dangerous to employ force in turning the child. The late Dr. Garthshore, being solicited by a patient to deliver under these circumstances, found the contraction so great, that the uterus was ruptured by the violence made use of, and the patient was thereby speedily destroyed.

Dr. Merriman's account of *plurality of births* is entertaining and wonderful; and if we had not extended this article beyond our limits, we should have extracted that part of the Appendix for the amusement of our readers.

A most valuable collection of cases is presented by our author, exemplifying the treatment of different kinds of *uterine hæmorrhage.* The concluding remarks on the improper use of opium should be read by every accoucheur.

A no less valuable collection of instances of labour, accompanied with *convulsions,* next attracts our notice. Puerperal convulsions have been supposed to arise from great tenderness and irritability of the os uteri. In speaking of one of the cases the author says,

"On the contrary, the *os uteri* allowed the finger to be passed through its aperture, and to be pressed in every direction with the greatest ease. I have found this to be the case in every instance." P. 269.

In one patient who died of convulsions the following post mortem appearances were found:

"The vessels of the brain were much loaded with blood; there was a very trifling effusion on the surface. At the very posterior part of the left lobe about a tea spoonful of blood was extravasated.

The *uterus* shewed no particular marks of disease, except that on the right side there was a slight appearance of inflammation or contusion." P. 273.

These appearances are sufficient to shew the propriety of adopting the plan of vigorous depletion, so strongly insisted on by the author. In our own patients, indeed, there has usually existed a previous, disturbed state of the mind, indicating, some time before labour has commenced, a congested condition of the vessels in the brain. On one occasion mental imbecility and hemiplegia preceded the confinement for the space of three months, and followed it for three weeks; and in one of Dr. Merriman's patients slight maniacal affections continued a long time after delivery.

Cases of inversion and of spontaneous inversion of the uterus are described with minuteness; we are next favoured with an account of the extirpation of an inverted uterus by Mr Chevalier; then with remarks on the fillet; next with a list of cases in which the Cæsarian operation has been performed in the British islands; and the Appendix closes with a specification of the deformities of the pelvis, and cases representing the effect of diet upon pregnant women.

Various excellent tables, pointing out the occurrences, extraordinary incidents, deaths, &c. to which the processes of pregnancy and parturition are liable, conclude the volume.

Our readers will, by this time, have formed a most favourable opinion of Dr. Merriman's Synopsis; but they must not be content with an analysis of a work, which it is impossible to epitomise. They must not fail to purchase it, as even the smallest medical library cannot be complete without it. The humble and economical shape of the work is no less entitled to our respect than the sentiments it contains; and we cannot too often repeat, that a due attention to this circumstance would, in every instance, be alike beneficial to the author, the bibliopolist, and the public.

The humanity of Dr. Merriman in a department, where so much is left to the mercy as well as judgment of the practitioner, is worthy the imitation of, and we hope is duly appreciated by, all his pupils; and we are happy to find him so deservedly elevated, as to possess the means of diffusing, among a great proportion of the profession, superior instruction, joined with such Christian qualities, as will always distinguish the medical philosopher from the unfeeling, unreflecting, and barbarous empiric.

